# Improving Self-Esteem With Motivational Quotes: Opportunities for Digital Health Technologies for People With Chronic Disorders

**DOI:** 10.3389/fpsyg.2018.02126

**Published:** 2018-11-02

**Authors:** Alisa Bedrov, Grzegorz Bulaj

**Affiliations:** ^1^Department of Psychology, Duke University, Durham, NC, United States; ^2^Department of Medicinal Chemistry, College of Pharmacy, University of Utah, Salt Lake City, UT, United States

**Keywords:** self-management, self-care, empowerment, digital therapeutics, mHealth, depression, anxiety

## Introduction

Living with a chronic medical condition is often accompanied by low self-esteem, a diminished sense of personal worth, and lower self-efficacy, a diminished sense of one's ability to influence behavioral outcomes. This can significantly affect health-related quality of life and wellbeing, with low self-esteem and self-efficacy contributing to poor coping, helplessness, a decrease in positive health behaviors, and increased risk of comorbidities. Thus, it becomes important to promote self-esteem and empowerment over one's condition.

Digital health technologies (also known as mobile health, mHealth, digital medicine, or digital therapeutics) have received increased recognition among chronically-ill patients and healthcare professionals as non-pharmacological treatment strategies for providing disease-specific, self-management content while also improving patient engagement. Since motivational quotes are increasingly used in various media and therapeutic programs to promote positive thinking and self-esteem, this article explores their medical applications and integration into digital health, with particular emphasis on tailoring different categories of motivational quotes to address specific causes of low self-esteem in chronically-ill adult populations.

## Self-esteem and self-efficacy in chronic conditions

Being afflicted with a chronic mental or physical condition, such as depression, diabetes, or other long-term illness, presents a myriad of difficulties that significantly impact quality of life. In addition to physical symptoms that interfere with daily activities, one faces a range of psychosocial difficulties. Common sources of distress include unmet emotional support needs, feelings of loss or alienation, and tolls on finances, work, and leisure (Vellenga and Christenson, 1994; Morasso et al., 1999; Vallis et al., 2016). Mental illness is frequently accompanied by depressed mood and anxiety, and chronic physical diseases such as asthma, arthritis, and diabetes have similarly been associated with comorbid depression (Moussavi et al., 2007; Connell et al., 2012). Another struggle for chronically-ill individuals is low self-esteem, which is both a consequence of the condition and obstacle for effective self-management and coping. Low self-esteem has been correlated with self-stigma that often accompanies mental illness, in which one internalizes negative perceptions of the condition and perceives the illness as a poor reflection of the self (Corrigan et al., 2006). It may also arise from the decreased independence and autonomy frequently associated with chronic illness, and from maladaptive coping strategies involving avoidance or blame (Felton et al., 1984; Connell et al., 2012). Low self-esteem in turn becomes a risk factor for depression, anxiety, eating disorders, aggression, and substance abuse, further compounding the negative effects of a chronic condition and introducing significant comorbidities (Mann et al., 2004; Sowislo and Orth, 2013). Thus, it becomes important to assist chronically-ill individuals with overcoming low self-esteem in order to prevent negative consequences on disease self-management and quality of life.

High self-esteem becomes even more critical given its potential impact on self-efficacy, which has been associated with reduced disease impact and more effective self-management through medication adherence, stress management, exercise, and nutrition (Clark et al., 1988; Clark and Dodge, 1999). Perceived self-efficacy has also been shown to reduce depressive symptoms and predict positive health behavior change (Strecher et al., 1986; Karademas, 2006). Several self-management programs emphasize self-efficacy for coping with illness, improving health status, and collaborating with health professionals to maximize treatment outcomes (Lorig et al., 2001; Bodenheimer et al., 2002). Self-efficacy can influence effective disease self-management by helping one persevere through difficulties, recover from setbacks, and maintain good habits (Bandura, 2004). Consequently, self-efficacy becomes essential for dealing with chronic disorders, for it is not uncommon for feelings of despair, hopelessness, or frustration to cause individuals to neglect positive health behaviors.

While self-esteem and self-efficacy are distinct constructs, high levels of both enable better coping with and recovery from physical diseases (Mann et al., 2004). Furthermore, self-esteem has been identified as an intrapersonal influence on self-efficacy, suggesting that increasing self-esteem could subsequently increase self-efficacy (Flay and Petraitis, 1994; Flay et al., 2009). According to the Theory of Triadic Influence, high self-esteem increases the value of willpower and self-determination in changing health-related behaviors, which in turn builds self-efficacy (Flay and Petraitis, 1994). Thus, improving low self-esteem could have downstream effects of increasing one's willpower to effectively manage the disease. Additionally, high self-esteem may prevent a chronically-ill individual from discounting positive feedback about his or her self-efficacy (Strecher et al., 1986). This would protect against falling into a negative, doubtful state regarding one's ability to cope with or overcome the illness. Given its relationship to self-esteem, one way to address the physical and psychosocial difficulties faced by chronically-ill individuals would be to improve self-efficacy by targeting low self-esteem.

## Addressing low self-esteem with motivational quotes

Motivational quotes have been integrated into various programs and interventions in clinical and educational settings, effectively increasing confidence, motivation, empowerment, and satisfaction in adults struggling with stress, anxiety, depression, mental illness, and substance abuse (Czuchry and Dansereau, 2005; Kendall et al., 2005; Brann and Sloop, 2006; Marchinko and Clarke, 2011; Littlechild et al., 2013; Poon, 2016). However, inspirational quotes or similar attempts to increase self-esteem are not always effective. Self-selected inspirational quotes had no effect on children's perceived autonomy, competence, or intrinsic motivation in the classroom (Collins, 2015), and inflated praise of children with low self-esteem led to decreased challenge seeking rather than increased self-esteem (Brummelman et al., 2014). Self-help literature in general has been criticized as overly simplistic and idealistic, with excessive optimism, self-affirmation, and imperative statements not always having the intended positive effects and sometimes leading to lower self-esteem or unrealistic world views (Gokhale, 2012). This suggests that some motivational quotes are more effective than others, with different types of quotes being better suited to address low self-esteem depending on its causes. Table 1 presents several categories of motivational quotes, each of which can be tailored to address a specific source of low self-esteem. By carefully selecting motivational quote categories for particular individuals, digital health therapies and interventions may more effectively improve low self-esteem in chronically-ill individuals. This article specifically focuses on motivational quotes for adult populations, but the same approach can apply to children by using more child-friendly inspirational quotes.

**Table 1:** Categories of motivational quotes for addressing low self-esteem.

**Quote type**	**Description**	**Example**
Choice	Presents the option of acting to change one's present state or maintaining the status quo, preserving the individual's sense of agency and control over steps taken to improve wellbeing	“The difference between misery and happiness depends on what we do with our attention.”—Sharon Salzberg “Instead of begging to be picked by others, you have the choice to pick yourself and build your brand.”—Bernard Kelvin Clive
Self-directed question	Poses a question to the individual that stimulates introspection and self-examination which in turn may promote insight into the self and the causes behind diminished wellbeing	“Why should we worry about what others think of us, do we have more confidence in their opinions than we do our own?”—Brigham Young “What would it be like to look in the mirror and actually accept what you see? Not loathe the reflection, or despite it, or be resigned to it? But to like it?”—Justina Chen
Cause-and-effect	Outlines the positive effects that may result from particular thoughts or actions, preserving the individual's sense of agency by only providing information rather than ordering action	“Once you start making the effort to ‘wake yourself up'—that is, be more mindful in your activities—you suddenly start appreciating life a lot more.”—Robert Biswas-Diener “We build confidence by daring to step outside our comfort zone in small increments.”—Sam Owen
Imperative/command	Provides direction and explicit commands for the individual to follow in order to improve wellbeing, potentially limiting personal agency but also providing valuable guidance	“Look within, and seek That.”—Jalaluddin Rumi “If you cannot do great things, do small things in a great way.”—Napoleon Hill
Empowerment/“You”	Addresses the individual directly with positive, affirming statements meant to empower and increase self-esteem, self-worth, self-confidence, etc.	“You yourself, as much as anybody in the entire universe, deserve your love and affection.”—Siddhartha Gautama “Stop trying to ‘fix' yourself; you're NOT broken! You are perfectly imperfect and powerful beyond measure.”—Steve Maraboli
Experience of others/“I”	Offers the opinion or first-person account of someone else, providing the individual with someone to relate to or an example of how others deal with diminished wellbeing or difficulties	“Why am I trying to be somebody? I am somebody.”—Eric Christopher Jackson “I am only one, but I am one. I cannot do everything, but I can do something. And because I cannot do everything, I will not refuse to do the something that I can do.”—Edward Everett Hale
Reality check	Disrupts the perception of a perfect world, highlighting life's negative aspects and showing the individual that it is natural to face pain and difficulties, emphasizing that others also share these experiences	“Even a happy life cannot be without a measure of darkness, and the word happy would lose its meaning if it were not balanced by sadness.”—Carl G. Jung “No one has it all, and no one lacks it all.”—Christopher Peterson
General statement	Discusses general concepts or situations in life, often in an observational manner, so as to guide cognitions, mindsets, or perspectives of the world to be more positive, thus promoting wellbeing without imposing on the individual	“Surrendering is not giving up—it is gaining strength.”—Grace Sara “Every person on this earth is full of great possibilities that can be realized through imagination, effort, and perseverance.”—Scott Barry Kaufman

*A more extensive list of motivational quotes organized by category can be found in the supplementary material under Datasheet 1*.

Low self-esteem is multi-faceted and may arise from various factors related to chronic illness. According to sociometer theory, self-esteem reflects the extent that one feels accepted by others and is a social construct that highly values perceptions of close friends and family (Leary and Baumeister, 2000). Living with a chronic disease often makes one feel isolated or excluded, whether it be from perceived disease-related stigma or missing out on social activities due to decreased mobility or high treatment demands. In this case, the best way to address low self-esteem is to situate the individual within a social context, allowing one to relate to others or receive direct feedback indicating understanding and social acceptance. Both “Experience of Others/‘I”' and “Empowerment/‘You”' motivational quotes would fulfill this purpose, with the former allowing one to relate to others' firsthand experiences, and the latter providing positive feedback and clear indication of social support.

However, individuals with low self-esteem have also preferred accurate self-appraisals over self-enhancing feedback, with depressed individuals holding more accurate self-views to determine whether behavioral changes are necessary (Kirkpatrick and Ellis, 2001). In this case, attempting to convince one of the faultiness of negative self-appraisals would be less efficient. Unrealistically positive appraisals could be met with skepticism, disgust, or rejection for being overly optimistic. A more effective strategy than “Empowerment/‘You”' quotes would be “Reality Check” or “General Statement” quotes that acknowledge the negative aspects of life one is experiencing, or discuss general concepts in a passive, observational manner, without presenting what could otherwise be perceived as false praise.

Low self-esteem may also arise from poor self-evaluation due to socially comparing oneself to others (Mann et al., 2004). With chronic illness, others can easily seem superior because they are not debilitated by the physical or psychological symptoms that the afflicted individual struggles with daily. This source of low self-esteem may be addressed in two ways. The first approach is to eliminate the perception of others as somehow superior. “Reality Check” quotes highlighting others' imperfections or difficulties would effectively disrupt the high esteem one holds others in, illustrating that they are not inherently better or flawless. The second approach is to make self-evaluations less deprecating and more affirming. “Empowerment/‘You”' and “Self-Directed Question” motivational quotes would encourage positive self-evaluations by emphasizing the individual's laudable qualities and promoting self-reflection that would subsequently allow one to recognize personal strengths and value.

Another cause of low self-esteem may be confusion about one's identity, leading to maladaptive coping responses of escapism or avoidance of current issues (Mann et al., 2004). In having a chronic medical condition, one may feel lost and not know one's place in the world, unable to separate the self from the disease, treatments, or hospital settings that seem to consume everyday life. At this point, the individual is likely to accept direction from others in searching for a solution to increase confidence and self-assurance. “Cause-and-Effect” and “Imperative/Command” motivational quotes would effectively provide this guidance, allowing one to take the necessary steps toward clarity and self-discovery.

In addressing low self-esteem, two important considerations are autonomy and perceived control, for chronically-ill individuals may be highly susceptible to perceiving a lack of control over symptoms or life in general. This may arise from the unpredictable nature of symptoms or important decisions being controlled by medical professionals rather than the individual. Adolescents with serious or chronic diseases were found to have a significantly reduced sense of control over their health-related futures (Kellerman et al., 1980), and control, autonomy, and choice regarding quality of life, symptoms, and major decisions were particularly important for individuals with mental health problems (Connell et al., 2012). Experiencing personal control over life was positively correlated with quality of life in late-stage cancer patients (Lewis, 1982). Thus, for individuals particularly concerned with maintaining control over their lives, and whose low self-esteem stems from a perceived lack of control or autonomy, “Choice” and “General Statement” motivational quotes would be most effective. These categories merely present general observations or options for action, allowing one to decide whether to act or not without imposing outside control or infringing upon one's sense of agency.

As the above discussion illustrates, the multi-faceted nature of self-esteem requires motivational quotes to be specifically tailored to the cause of low self-esteem to be effective, rather than applied indiscriminately across all situations.

## Digital health and mobile medical applications

Although still in its infancy, digital medicine is being embraced by patients, doctors, nurses, healthcare and pharmaceutical industries, and regulatory agencies such as the US Food and Drug Administration (FDA). Several FDA-approved mobile medical apps (for diabetes and addiction) and video games (for stroke and traumatic brain injury) illustrate how non-pharmacological interventions delivered by digital technologies can provide clinical benefits for chronically-ill patients. Positive findings in recent clinical studies of mobile health interventions for depression (Mantani et al., 2017; Ben-Zeev et al., 2018; Berger et al., 2018; Silva Almodovar et al., 2018) emphasize new opportunities to improve treatments of mental disorders.

Digital therapies can deliver behavioral interventions by mobile apps, internet (web-based apps), and video games. An apparent benefit of mobile health is its “personalized medicine” aspect that adjusts digital content to an individual's current needs through just-in-time adaptive interventions (Nahum-Shani et al., 2018). The use of wearables can further optimize digital interventions through neurobiofeedback mechanisms (Ramirez et al., 2015), with new biofeedback technologies, such as the FDA-cleared smartwatch Embrace (Empatica), being developed. The flexibility of digital content in mobile health technologies is further emphasized by adding music as an “active component” of therapy (Schriewer and Bulaj, 2016; Afra et al., 2018) or integrating exercise and educational empowerment into a video game designed for children with cancer (Bruggers et al., 2018). Integrating judiciously tailored motivational quotes into digital content as an “active ingredient” in mobile and web-based apps may offer additional means to improve patient engagement, self-esteem, and ultimately self-efficacy. Daily delivery of such motivational quotes will likely appeal to chronically-ill individuals across various disorders, for many of them live with depression or anxiety as comorbidities.

In conclusion, digital health technologies are promising for delivering self-esteem interventions to improve engagement and treatment outcomes and ultimately improve quality of life for people living with chronic disorders.

## Author contributions

AB and GB conceived the study and wrote and edited the manuscript. AB reviewed the literature, compiled, and analyzed the quotes.

### Conflict of interest statement

GB is a co-founder, officer, and board member in Epicadence, Public Benefit Corporation, focused on development of mobile software for epilepsy patients. GB is a co-inventor on patent-pending Multimodal Platform for Treating Epilepsy licensed to Epicadence PBC. GB is a co-inventor on two issued US patents 9,569,562 and 9,747,423 Disease Therapy Game Technology. These patents are related to the exercise-empowerment video game technology and are owned by the University of Utah. The remaining author declares that the research was conducted in the absence of any commercial or financial relationships that could be construed as a potential conflict of interest.

## References

[B1] Afra P. Bruggers C. S. Sweney M. Fagatele L. Alavi F. Greenwald M.. (2018). Mobile software as a medical device (SaMD) for the treatment of epilepsy: development of digital therapeutics comprising behavioral and music-based interventions for neurological disorders. Front. Hum. Neurosci. 12:171. 10.3389/fnhum.2018.00171 29780310 PMC5946004

[B2] Bandura A. (2004). Health promotion by social cognitive means. Health Educ. Behav. 31, 143–164. 10.1177/1090198104263660 15090118

[B3] Ben-Zeev D. Brian R. M. Jonathan G. Razzano L. Pashka N. Carpenter-Song E.. (2018). Mobile health (mHealth) versus clinic-based group intervention for people with serious mental illness: a randomized controlled trial. Psychiatr. Serv. 69, 978–985. 10.1176/appi.ps.201800063 29793397

[B4] Berger T. Krieger T. Sude K. Meyer B. Maercker A. (2018). Evaluating an e-mental health program (“deprexis”) as adjunctive treatment tool in psychotherapy for depression: Results of a pragmatic randomized controlled trial. J. Affect. Disord. 227, 455–462. 10.1016/j.jad.2017.11.021 29154168

[B5] Bodenheimer T. Lorig K. Holman H. Grumbach K. (2002). Patient self-management of chronic disease in primary care. JAMA 288, 2469–2475. 10.1001/jama.288.19.2469 12435261

[B6] Brann D. W. Sloop S. (2006). Curriculum development and technology incorporation in teaching neuroscience to graduate students in a medical school environment. Adv. Physiol. Educ. 30, 38–45. 10.1152/advan.00068.2005 16481608

[B7] Bruggers C. S. Baranowski S. Beseris M. Leonard R. Long D. Schulte E.. (2018). A prototype exercise-empowerment mobile video game for children with cancer, and its usability assessment: developing digital empowerment interventions for pediatric diseases. Front. Pediatr. 6:69. 10.3389/fped.2018.00069 29686977 PMC5900044

[B8] Brummelman E. Thomaes S. Orobio de Castro B. Overbeek G. Bushman B. J. (2014). “That's not just beautiful – that's incredibly beautiful!”: the adverse impact of inflated praise on children with low self-esteem. Psychol. Sci. 25, 728–735. 10.1177/0956797613514251, 24434235

[B9] Clark N. M. Dodge J. A. (1999). Exploring self-efficacy as a predictor of disease management. Health Educ. Behav. 26, 72–89. 10.1177/109019819902600107 9952053

[B10] Clark N. M. Rosenstock I. M. Hassan H. Evans D. Wasilewski Y. Feldman C. (1988). The effect of health beliefs and feelings of self efficacy on self management behavior of children with a chronic disease. Patient Educ. Couns. 11, 131–139. 10.1016/0738-3991(88)90045-6

[B11] Collins G. (2015). Language to motivate learning: the power of inspirational quotes. A Rising Tide. 7, 1-22. Available online at: http://www.smcm.edu/mat/wp-content/uploads/sites/73/2015/06/Gabrielle-Collins-2015.pdf

[B12] Connell J. Brazier J. O'Cathain A. Lloyd-Jones M. Paisley S. (2012). Quality of life of people with mental health problems: a synthesis of qualitative research. Health Qual. Life Outcomes. 10:138. 10.1186/1477-7525-10-138 23173689 PMC3563466

[B13] Corrigan P. W. Watson A. C. Barr L. (2006). The self-stigma of mental illness: implications for self-esteem and self-efficacy. J. Soc. Clin. Psychol. 25, 875–885. 10.1521/jscp.2006.25.8.875

[B14] Czuchry M. Dansereau D. F. (2005). Using motivational activities to facilitate treatment involvement and reduce risk. J. Psychoactive Drugs 37, 7–13. 10.1080/02791072.2005.10399744 15916247

[B15] Felton B. J. Revenson T. A. Hinrichsen G. A. (1984). Stress and coping in the explanation of psychological adjustment among chronically ill adults. Soc. Sci. Med. 18, 889–898. 10.1016/0277-9536(84)90158-8. 6729517

[B16] Flay B. R. Petraitis J. (1994). The theory of triadic influence: a new theory of health behavior with implications for preventive interventions. Adv. Med. Sociol. 4, 19–44.

[B17] Flay B. R. Snyder F. J. Petraitis J. (2009). The theory of triadic influence in Emerging Theories in Health Promotion Practice and Research, eds DiClemente R. J. Crosby R. A. Kegler M. C. (San Francisco, CA: Jossey-Bass), 451–510.

[B18] Gokhale M. (2012). The implications of simplification in “inspirational literature”. International J. Soc. Sci. Hum. 2, 400–404. 10.7763/IJSSH.2012.V2.134

[B19] Karademas E. C. (2006). Self-efficacy, social support and well-being: the mediating role of optimism. Pers. Individ. Dif. 40, 1281–1290. 10.1016/j.paid.2005.10.019

[B20] Kellerman J. Zeltzer L. Ellenberg L. Dash J. Rigler D. (1980). Psychological effects of illness in adolescence. I. Anxiety, self-esteem, and perception of control. J. Pediatr. 97, 126–131. 10.1016/S0022-3476(80)80152-1 6966685

[B21] Kendall J. Waddington C. Kendall C. (2005). The daily moment: a stress reduction program for cancer center staff. J. Oncol. Manage. 14, 68–71. 16161643

[B22] Kirkpatrick L. A. Ellis B. J. (2001). An evolutionary-psychological approach to self-esteem: multiple domains and multiple functions in Blackwell Handbook of Social Psychology: Interpersonal Processes, eds Fletcher G. J. O. Clark M. S. (Malden, MA: Blackwell Publishing), 411–436.

[B23] Leary M. R. Baumeister R. F. (2000). The nature and function of self-esteem: sociometer theory. Adv. Exp. Soc. Psychol. 32, 1–62. 10.1016/S0065-2601(00)80003-9

[B24] Lewis F. M. (1982). Experienced personal control and quality of life in late-stage cancer patients. Nurs. Res. 31, 113–119. 10.1097/00006199-198203000-00012 6926649

[B25] Littlechild B. Smith A. H. Meredith-Windle G. Gale T. Lloyd M. Hawley C. (2013). Recovery approaches in mental health: a qualitative evaluation of the whole life therapy programme for persons with schizophrenia. Health 5, 582–587. 10.4236/health.2013.53A077

[B26] Lorig K. R. Sobel D. S. Ritter P. L. Laurent D. Hobbs M. (2001). Effect of a self-management program on patients with chronic disease. Effect. Clin. Pract. 4, 256–262. Available online at: http://ecp.acponline.org/novdec01/lorig.pdf 11769298

[B27] Mann M. Hosman C. M. Schaalma H. P. De Vries N. K. (2004). Self-esteem in a broad-spectrum approach for mental health promotion. Health Educ. Res. 19, 357–372. 10.1093/her/cyg041 15199011

[B28] Mantani A. Kato T. Furukawa T. A. Horikoshi M. Imai H. Hiroe T.i.. (2017). Smartphone cognitive behavioral therapy as an adjunct to pharmacotherapy for refractory depression: randomized controlled trial. J. Med. Internet Res. 19:e373. 10.2196/jmir.8602 29101095 PMC5695656

[B29] Marchinko S. Clarke D. (2011). The wellness planner: empowerment, quality of life, and continuity of care in mental illness. Arch. Psychiatr. Nurs. 25, 284–293. 10.1016/j.apnu.2010.10.003 21784286

[B30] Morasso G. Capelli M. Viterbori P. Di Leo S. Alberisio A. Costantini M.. (1999). Psychological and symptom distress in terminal cancer patients with met and unmet needs. J. Pain Symptom Manage. 17, 402–409. 10.1016/S0885-3924(99)00034-2 10388245

[B31] Moussavi S. Chatterji S. Emese V. Tandon A. Patel V. Ustun B. (2007). Depression, chronic diseases, and decrements in health: results from the world health surveys. Lancet 370, 8–14. 10.1016/S0140-6737(07)61415-9 17826170

[B32] Nahum-Shani I. Smith S. N. Spring B. J. Collins L. M. Witkiewitz K. Tewari A.. (2018). Just-in-time adaptive interventions (JITAIs) in mobile health: key components and design principles for ongoing health behavior support. Ann. Behav. Med. 52, 446–462. 10.1007/s12160-016-9830-8 27663578 PMC5364076

[B33] Poon S. K. (2016). *Pacifica*: stressed or worried? An app to help yourself (Mobile App User Guide). Br. J, Sports Med. 50, 191–192. 10.1136/bjsports-2015-095747

[B34] Ramirez R. Palencia-Lefler M. Giraldo S. Vamvakousis Z. (2015). Musical neurofeedback for treating depression in elderly people. Front. Neurosci. 9:354. 10.3389/fnins.2015.00354 26483628 PMC4591427

[B35] Schriewer K. Bulaj G. (2016). Music streaming services as adjunct therapies for depression, anxiety, and bipolar symptoms: convergence of digital technologies, mobile apps, emotions, and global mental health. Front. Public Health 4:217. 10.3389/fpubh.2016.00217 27747209 PMC5043262

[B36] Silva Almodovar A. Surve S. Axon D. R. Cooper D. Nahata M. C. (2018). Self-directed engagement with a mobile app (Sinasprite) and its effects on confidence in coping skills, depression, and anxiety: retrospective longitudinal study. JMIR mHealth uHealth 6:e64. 10.2196/mhealth.9612 29549066 PMC5878360

[B37] Sowislo J. F. Orth U. (2013). Does low self-esteem predict depression and anxiety? A meta-analysis of longitudinal studies. Psychol. Bull. 139, 213–240. 10.1037/a0028931 22730921

[B38] Strecher V. J. DeVellis B. M. Becker M. H. Rosenstock I. M. (1986). The role of self-efficacy in achieving health behavior change. Health Educ. Behav. 13, 73–92. 10.1177/109019818601300108 3957687

[B39] Vallis M. Bruns K. K. Hollahan D. Ross S. Hahn J. (2016). Diabetes attitudes, wishes, and needs second study (DAWN2): understanding diabetes-related psychosocial outcomes for Canadians with diabetes. Can. J. Diab. 40, 234–241. 10.1016/j.jcjd.2015.11.002 26948024

[B40] Vellenga B. A. Christenson J. (1994). Persistent and severely mentally ill clients' perceptions of their mental illness. Issues Ment. Health Nurs. 15, 359–371. 10.3109/01612849409006914 8056567

