# Supplementary material for: Improving Self-Esteem With Motivational Quotes: Opportunities for Digital Health Technologies for People With Chronic Disorders
Source: Front Psychol. 2018 Nov 2;9:2126. doi: 10.3389/fpsyg.2018.02126 (PMC6224439; doi:10.3389/fpsyg.2018.02126)
Supplement: Supplementary file 1 [file Data_Sheet_1.PDF]

frontiers

Supplementary Material

Improving Self-Esteem with Motivational Quotes: Opportunities for Digital Health Technologies for People with Chronic Disorders

Alisa Bedrov*, Grzegorz Bulaj

*** Correspondence:** Alisa Bedrov: alisa.bedrov@duke.edu

1 Supplementary Data

The following is a compilation of quotes categorized by types of quotes. The sorting of these quotes was done subjectively and is not strictly defined. There are many quotes which may fall into multiple categories and serve multiple purposes, thus being effective for multiple sources of low self-esteem.

Choice: *presents the option of acting to change one's present state or maintaining the status quo, preserving the individual's sense of agency and control over steps taken to improve wellbeing*

"The difference between misery and happiness depends on what we do with our attention." – Sharon Salzberg

"The difference between a life laced through with frustration and one sustained by happiness depends on whether it is motivated by self-hatred or by real love for oneself." – Sharon Salzberg

"Instead of begging to be picked by others, you have the choice to pick yourself and build your brand." – Bernard Kelvin Clive

"We can choose to dampen hopes, thus preserving a current order (if almost everything is determined by biology, why try?), or we kindle the flames of hope and become agents of change (if anything might be affected by culture, why *not* try?)." – Gloria Steinem

"Whether pleasure or pain, every situation in your life serves a purpose. It is up to us to recognize what that purpose could be." – Steve Maraboli

"Loving yourself costs nothing, but not loving yourself could cost you everything." – B.D. Jordan

"Just because you have baggage doesn't mean you have to lug it around." – Richie Norton

"You do not need to pay attention to those voices within you that create pain, or make you feel less competent, smart or able." – Sanaya Roman

"Depression is a prison. For some it is easy and short, for others it is difficult and long. For some the days go by easier than most and for others the days are eternity. But it is your choice to try and escape." – C. Shultz

---

Self-Directed Question: *poses a question to the individual that stimulates introspection and self-examination which in turn may promote insight into the self and the causes behind diminished wellbeing*

"Why should we worry about what others think of us, do we have more confidence in their opinions than we do our own?" – Brigham Young

"What would it be like to look in the mirror and actually accept what you see? Not loathe the reflection, or despite it, or be resigned to it? But to like it?" – Justina Chen

"How would your life be different if…you stopped allowing other people to dilute or poison your day with their words or opinions?" – Steve Maraboli

"What do you see when you look in the mirror? I hope it goes beyond beauty. I hope what you see is that person who's worth it and deserves nothing less than the best, I hope that you see beyond the pain, I hope that you see the glory ahead of you[…]" – Temitayo Olami

"What is the feeling tone in your life that radiates in you and makes you shine, that makes you feel whole, that makes you feel your heart? That feeling tone, which we long to hear from others, is the tone we want to practice with ourselves. That's where we want to live with ourselves." – Alexandra Katehakis

"How could you expect people to treat you well, when you don't treat yourself right?" – Rafaa Khiari

"Why wait for later when there might not be a later? Why wait for next time when there might not be a next time? We all have just today to express our love." – Ron Baratono

"[…] the Good, the Bad and the Ugly are present in all of us. What determines which one dominates is the one that is fed. Which one are you feeding?" – Bobby F. Kimbrough Jr.

Cause-and-Effect: *outlines the positive effects that may result from particular thoughts or actions, preserving the individual's sense of agency by only providing information rather than ordering action*

"Once you start making the effort to 'wake yourself up' – that is, be more mindful in your activities – you suddenly start appreciating life a lot more." – Robert Biswas-Diener

"We build confidence by daring to step outside our comfort zone in small increments." – Sam Owen

"The secret to life is to have no fear. When you can let go of what others think about you, how something is going to turn out, or how your past will affect your future, then you are finally living life free." – Shannon L. Alder

“There is a silent beauty hidden amongst pain; if you sit in it you may never find it; if you grow through it, you’ll find the treasure.” – Nikki Rowe

“Taking charge of our life reinforces our self-worth.” – Sam Owen

“Be content with what you have. Rejoice in how things are. When you realize there is nothing lacking, the whole world belongs to you.” – Lao Tzu

“When we are open to new possibilities, we find them. Be open and skeptical of everything.” – Todd Kashdan

“What we plant in the soil of contemplation, we shall reap in the harvest of action.” – Meister Eckhart

“You will be your best self when you take time to understand what you really need, feel and want.” – Deborah Day

“Acknowledgement is possession. When you acknowledge, think or have conviction in something, it actually will come true.” – Stephen Richards

“If we can acquire an attitude of self-belief, then we will surely determine our future actions and our future life opportunities.” – Stephen Richards

“When you are in alignment with your life purpose, there will be a knowing in your heart that is so strong, that it will let you know your true calling. This will provide you with all the energy you need to fulfill your mission.” – Renae A. Sauter

“When we try to find the good in ourselves and others, we pull those good traits up out of hiding and keep them alive, versus pulling out our bad qualities.” – Emilyann Girdner

“The more confident and appreciative of who you are, the less hold depression has over you.” – Omoakhuana Anthonia

“There are many ways your intuition can get a message to you. You will start to pick them up once you learn to quiet the mental chatter.” – Stevie Puckett

“It’s when you feel low about yourself, others actions and opinions will affect you. When you look at yourself as a confident person, it’s then; you’ll find the most peace and understanding, that it’s your world you have to live with, not theirs.” – Ron Baratono

“It’s nice to begin each day with an open mind but a wonderful fulfillment will come with an open heart.” – Ron Baratono

“When you can picture a happier life, you will be determined to work at the things that prevent it from happening.” – Keysha Jade

“If we can write or sing or create in some way, even when we are dealing with difficulties or pain, then it becomes something bigger than ourselves – and often beautiful.” – Brenda Peterson

“Believe in yourself and the world will follow suit.” – Tadahiko Nagao

“Accepting personal responsibility for your life frees you from outside influences – increases your self-esteem – boosts confidence in your ability to decisions – and ultimately leads to achieve success in life.” – Roy T. Bennett

“Discussing positive experiences leads to heightened well-being, increased overall life satisfaction and even more energy.” – Nathaniel Lambert

“Daily dance uplift the soul to spiritual realms.” – Lailah Gifty Akita

“As you become more aligned with the truth of who you are, the question of liking yourself goes away. It is a natural state of being.” – Rachel Archelaus

“Beliefs that are good promote your potential and enhance your unique special qualities.” – Deborah Day

“The goal is to learn to be nurturing with yourself so you can feel free.” – Deborah Day

“Keeping busy and making optimism a way of life can restore your faith in yourself.” – Lucille Ball

“If you celebrate your differentness, the world will, too. It believes exactly what you tell it – through the words you use to describe yourself, the actions you take to care for yourself, and the choices you make to express yourself. Tell the world you are one-of-a-kind creation who came here to experience wonder and spread joy. Expect to be accommodated.” – Victoria Moran

“Positive thinking is powerful thinking. If you want happiness, fulfillment, success and inner peace, start thinking you have the power to achieve those things. Focus on the bright side of life and expect positive results.” – Germany Kent

“Building confidence comes from overcoming the voice in your head that says you are not capable; silence the noise and then prove it wrong.” – Sam Owen

“Believe in yourself. Under-confidence leads to a self-fulfilling prophecy that you are not good enough for your work.” – Roopleen

“If you fall in darkness, there will be light. If your fall enter depression, there is some sort of happiness. Fall but get up. Weak but be strong. Life is just beginning.” – Claire Lunette

“Life is like a game of chess. To win you have to make a move. Knowing which move to make comes with insight and knowledge, and by learning the lessons that are accumulated along the way. We become each and every piece within the game called life!” – Allan Rufus

“Self-esteem is the switch in the circuit of your life that dims or brightness of your future. Bring it low and you don’t shine your light; raise it up and you brighten the corner where you are.” – Israelmore Ayivor

“With strong self-esteem you can climb any mountain and weather any storm.” – Joanne Madeline Moore

“If you change the way you look at things, the things you look at change.” – Wayne Dyer

“Defeating depression is like playing the carnival game Whac-a-mole. You have to give it your all and be on target to beat that sucker down when it pops up again and again. If you pay attention, learn from your past efforts, and keep at it, you can win.” – Mel Edwards

“One day, you will stand at the summit of a figurative mountain and look back on your life’s journey. And, to your utter amazement, you will see how your experiences with depression, dark and painful as they were, only added to the overall beauty of your life.” – Seth Adam Smith

“You can overcome whatever is going on around you if you believe in the light that lives within you.” – Justine Edward

“Until you value yourself, you won’t value your time. Until you value your time, you will not do anything with it.” – M. Scott Peck

---

Imperative/Command: *provides direction and explicit commands for the individual to follow in order to improve wellbeing, potentially limiting personal agency but also providing valuable guidance*

“Look within, and seek That.” - Jalaluddin Rumi

“If you cannot do great things, do small things in a great way.” – Napoleon Hill

“Follow your dreams. Figure out where you’d like to be, what you would like to be doing, and then figure out what you need to do to get there.” – Kennon M. Sheldon

“Be faithful to that which exists within yourself.” – André Gide

“Make peace with yourself before you move forward. Accept yourself, love yourself, and keep moving forward.” – Roy T. Bennett

“Be nice to yourself…It’s hard to be happy when someone is mean to you all the time.” – Christine Arylo

“Reflect what you want. Your light, your beauty, and your strength. Aspire for greatness – reflect who you are; not which deficits you maintain. Showcase the hidden treasures.” – Tiffany Luard

“Dare to be different. Find the courage to accept that you may always fit in, and the peace within to stop looking for the approval of others. Just be the amazing being that you are. People who shine the light of who they truly are, are inspiring to others.” – Eileen Anglin

“Pain in your past will become power in your future. So make suffering essential.” – Debasish Mridha

“Raise your awareness and cultivate your uniqueness.” – Amit Ray

“Speak positive affirmations into fruition. For instance, if you feel frustrated or anxious, you can say, “I am calm, and I can work through my situation to arrive at a positive outcome,” or “I am at peace with all things in my life, and success finds me easily.” – Tisha Marie Payton

“Set out to be the person you know you can be. Thinking about it, and talking about it is the first step. If you can imagine it for yourself then you can do it. Take action!” – Ron Baratono

“Always think and always dream. Wonder what’s around the next corner for yourself, and don’t be afraid to go there, but most of all Love. Loving yourself and the people around you is the easiest path through life. It will bring you around that corner, and places you’ve never been.” – Ron Baratono

“Let your pain lead to creativity.” – Sunday Adelaja

“Do not take life too seriously. You will never get out of it alive.” – Elbert Hubbard

“When making your choice in life, do not neglect to live.” – Samuel Johnson

“Embrace the pain to inherit the gain.” – Habeeb Akande

“Measure yourself by your best moments, not by your worst. We are too prone to judge ourselves by our moments of despondency and depression.” – Robert Johnson

“Love yourself and that is all the love you need.” – Amit Abraham

“Make not your thoughts your prisons.” – William Shakespeare

“Claim your success. The moment you doubt is the moment you undermine it.” – E.B. Davis

“Have the courage to love yourself like you always wished someone would.” – Vironika Tugaleva

“Permit no one to define you by your mistakes or your past.” – Bernard Kelvin Clive

“Each person has got a voice inside them. Communicate with it and take hold of it. Do not let it push and shove you around – you are its master!” – Stephen Richards

“Remember that there is nothing stable in human affairs; therefore avoid undue elation in prosperity, or undue depression in adversity.” – Socrates

“Dare to love yourself
as if you were a rainbow
with gold at both ends.” – Aberjhani

“Out of clutter, find simplicity.” – Albert Einstein

“If something happens that causes you Pain, remind yourself that you can’t get the Rainbow without a little Rain.” – *R.V.M.*

“Do not wait for extraordinary circumstances to do good; try using ordinary situations.” – Jean Paul Richter

“Decide what you stand for. And then stand for it all the time.” – Clayton Christensen

“Dwell on the beauty of life. Watch the stars, and see yourself running with them.” – Marcus Aurelius

“Smile, breathe and go slowly.” – Thich Nhat Hanh

“Love what you do and do what you love.” – Ray Bradbury

Empowerment/“You”: *addresses the individual directly with positive, affirming statements meant to empower and increase self-esteem, self-worth, self-confidence, etc.*

“You yourself, as much as anybody in the entire universe, deserve your love and affection.” – Siddhartha Gautama

“Stop trying to ‘fix’ yourself; you’re NOT broken! You are perfectly imperfect and powerful beyond measure.” – Steve Maraboli

“You stand strong in the truth of your beauty and journey through your day without attachment to the validation of others.” – Steve Maraboli

“Do not allow yourself or others to be defined by your limitations, but rather, abilities.” – Eric Duquette

“The most creative act you will ever undertake is the act of creating yourself.” – Deepak Chopra

“We all have special attributes that are of value to someone. Find your place of value. You are never useless. You just need to find your place.” – Innocent Mwatsikesimbe

“The bottom line is, what defines you isn’t how many times you crash, but the number of times you get back on the bike. As long as it’s one more, you’re all good.” – Sarah Dessen

“You may be the only person left who believes in you, but it’s enough. It takes just *one star* to pierce a universe of darkness. Never give up.” – Richelle E. Goodrich

“You were not meant for a mundane or mediocre life!” – Steve Maraboli

“At the core of your heart, you are perfect and pure. No one and nothing can alter that.” – Amit Ray

“Your value is in your individuality.” – Dorla Rudd Jenkins

“You are greater than you can possibly imagine, if you would only free yourself up to imagine.” – Craig D. Lounsbrough

“The important thing to remember…is that you are a human being and worthy of respect.” – Sarah Dessen

“Never forget that there is a soul within you, which loves you very much, only because it’s you.” – Munia Khan

“You are amazing! That is your secret.” – Debasish Mridha

“The most empowering, important belief is the belief in yourself, your capabilities, your strength, your choice, and your most infinite, most divine, most beautiful worth.” – Connie Kerbs

"Nourishing yourself in a way that helps you blossom in the direction you want to go is attainable, and you are worth the effort." – Deborah Day

"Nobody can treat you like a liability without your approval." – Ane Krstevska

"Rewriting the negative beliefs you have learned is the essence of becoming the director of your life." – Deborah Day

"You don't need confirmation from the world or another person that you matter. You simply do matter. When you finally believe that truth and live it then you can do amazing things with your life!" – Shannon L. Alder

"Treat yourself like someone who is amazingly special. You are special!" – Debasish Mridha

"Remember, you have been criticizing yourself for years and it hasn't worked. Try approving of yourself and see what happens." – Louise L. Hay

"In your own life it's important to know how spectacular you are." – Steve Maraboli

"You're always with yourself, so you might as well enjoy the company." – Diane Von Furstenberg

"When *you* dance, the whole universe dances." – Jalaluddin Rumi

"You have a unique gift to offer this world. Be true to yourself, be kind to yourself, read and learn about everything that interests you and keep away from people who bring you down. When you treat yourself kindly and respect the uniqueness of those around you, you will be giving this world an amazing gift…YOU!" – Steve Maraboli

"You are the most important person in your life. Your relationship with yourself defines your relationships with every other person you encounter. Celebrate yourself and celebrate all." – Avina Celeste

"Self-respect, self-worth and self-love, all start with self. Stop looking outside of yourself for your value." – Rob Liano

"You have the power to heal your life, and you need to know that. We think so often that we are helpless, but we're not. We always have the power of our minds…Claim and consciously use your power." – Louise L. Hay

"Your life, your achievement, your happiness, your person are of paramount importance. Live up to your highest vision of yourself no matter what the circumstances you might encounter." – Ayn Rand

"You don't have to be like anyone else. Just learn more about your own creative self and start blooming." – Deborah Day

"Courage is never to let your actions be influenced by your fears." – Arthur Koestler

"A big part of dealing with depression is realizing that you are in control of your own happiness." – Brian Michael Good

“If you can't learn to love yourself for yourself (and how could you with such a paradoxical motivation?)... then do it for us. Do it for the world. We need you. We need your mind. We need your attention. We need your thoughts.
Change your focus, and you will (not can, but will) change the world. You already matter. You just have to realize it for yourself.” – Vironika Tugaleva

“Be kind to yourself. Remember that when you abuse yourself, you will experience the anger, regret, and apathy of the bully as well as the depression, anxiety, and insecurity of the victim. Whatever you do, be kind to yourself.” – Vironika Tugaleva

“Loving yourself isn't just about celebrating your accomplishments and nurturing your talents. Those things are nice, sure. But that's not how we know others love us. We know others love us when they see us with our face on the ground, crying and weak, feeling like we've got nothing to offer the world—and they smile, and they reach out, and they love us anyway. Loving yourself is what you do when you fail, when you don't know, when you screw up, when you forget, when you lose everything. Loving yourself is what you do when you can't approve of what you've done. Loving yourself is what you do when you're not sure if it's going to get better. Loving yourself is what you must do in those moments when you can't like yourself. Real love is when you reach out for no good reason at all, except to love.” – Vironika Tugaleva

“[…] do this for me, for yourself, today—celebrate. Just like you'd celebrate a birthday or a graduation, celebrate your endless journey of self-discovery. You deserve it. You need it. We all do.” – Vironika Tugaleva

“Every great dream begins with a dreamer. Always remember, you have within you the strength, the patience, and the passion to reach for the stars to change the world.” – Harriet Tubman

“Many of us spend our whole lives running from feeling with the mistaken belief that you cannot bear the pain. But you have already borne the pain. What you have not done is feel all you are beyond that pain.” – Kahlil Gibran

“Life can be hard at times. People can be hard on each other. Being hard on yourself is completely unnecessary.” – Ron Baratono

“The more you try, so desperately, to prove a point to get someone’s approval, the more fleeting that approval might be, causing a chain of depreciation of self-esteem. Sometimes, all you need to do is truly love yourself and get only the approval and acceptance of the inner self.” – Ufuoma Apoki

“There is no way to genuinely, powerfully, truly love yourself while crafting a mask of perfection.” – Vironika Tugaleva

“The greatest thing about our times is that you don’t need permission to express yourself the way you wish.” – Nuno Roque

“It is hard to love yourself if you never spend time with yourself. ‘Alone Time’ is necessary.” – Izey Victoria Odiase

“Self-love is not the process of ignoring your flaws. Self-love is expanding your awareness to include your flaws and your strengths.” – Vironika Tugaleva

"Vulnerability really means to be strong and secure enough within yourself that you are able to walk outside without your armor on. You are able to show up in life as just you. That is genuine strength and courage. Armor may look tough, but all it does is mask insecurity and fear." – Alaric Hutchinson

"When you're different, sometimes you don't see the millions of people who accept you for what you are. All you notice is the person who doesn't." – Jodi Picoult

---

Experience of Others/"I": *offers the opinion or first-person account of someone else, providing the individual with someone to relate to or an example of how others deal with diminished wellbeing or difficulties*

"Why am I trying to be somebody? I am somebody." – Eric Christopher Jackson

"I am only one, but I am one. I cannot do everything, but I can do something. And because I cannot do everything, I will not refuse to do the something that I can do." – Edward Everett Hale

"It is not fit that I should give myself pain, for I have never intentionally given pain even to another." – Marcus Aurelius

"I have been in the shadows trying to hide from all my pain, but I realize that one day I will walk out the shadows with my head held high." – Leian Joseph

"Whenever I am in a difficult situation where there seems to be no way out, I think about all the times I have been in such situations and say to myself, 'I did it before, so I can do it again.'" – Idowu Koyenikan

"What in the world would ever lead me to believe that life is a series of opportunities that are readily available to everyone else but me? What really leads me to believe such an atrocious lie is that I don't believe in myself sufficiently to engage those opportunities in the first place." – Craig D. Lounsbrough

"Today I must look in the mirror and be thankful for the person who I find staring back at me." – Craig D. Lounsbrough

"When the world does its level best to devalue me in ways that are nothing short of brutal, all it does is evidence my value. For why would it expend such massive amounts of energy attempting to destroy something that's not there?" – Craig D. Lounsbrough

"It feels good not to think about anything that wears on me. We all need that kind of room in our lives." – Ron Baratono

"Wanting to be someone else is a waste of the person you are. I'd rather be hated for who I am, than loved for who I am not." – Kurt Cobain

"As long as I can see, hear, feel, think, change, grow and behave, I have great possibility. I'm going to take those risks and those possibilities, and I am going to grow and love and be and celebrate. I am worth it." – Sharon Wegschieder-Cruse

“When I let go of what I am, I become what I might be.” – Lao Tzu

“I said Somebody should do something about that. Then I realized I am somebody.” – Lily Tomlin

---

Reality Check: *disrupts the perception of a perfect world, highlighting life’s negative aspects and showing the individual that it is natural to face pain and difficulties, emphasizing that others also share these experiences*

“Even a happy life cannot be without a measure of darkness, and the word happy would lose its meaning if it were not balanced by sadness.” – Carl G. Jung

“No one has it all, and no one lacks it all.” – Christopher Peterson

“Flowers are not made by singing ‘Oh, how beautiful,’ and sitting in the shade.” – Rudyard Kipling

“The truth is, bad things don’t affect us as profoundly as we expect them to. That’s true of good things, too. We adapt very quickly to either.” – Daniel Gilbert

“Life isn’t about having, it’s about being…The ‘If I just had X, I would be happy’ syndrome is a mass delusion. When you look for happiness in mere objects, they are never enough. Look around. Look within.” – Nick Vujicic

“There is no magic cure, no making it all go away forever. There are only small steps upward; an easier day, an unexpected laugh, a mirror that doesn’t matter anymore.” – Laurie Halse Anderson

“Be real. Embrace that you have weakness. Because everyone does. Embrace that your body is not perfect. Because nobody’s is. Embrace that you have things you can’t control. We all have a list of them.” – Dan Pearce

“It’s probably unfair to expect the world at large, or even most people, to see us for all we are. It is essential, however, that we see ourselves for all we are.” – Victoria Moran

“Winning doesn’t necessarily mean you’ll walk away with a trophy. Sometimes winning means you dared to show up to all to see the shocked look of your rivals.” – Donavan Nelson Butler

“There is no quick fix-all procedure to heal your insecurities. Like any relationship, the relationship with yourself must be built and re-built one kind, loving conversation at a time.” – Vironika Tugaleva

“A happy person is not without sorrow or grief. Happiness is the acceptance of pain, not the lack of it.” – Vironika Tugaleva

“You may encounter defeats, but you must not be defeated.” – Maya Angelou

“We grow neither better nor worse as we get old, but more like ourselves.” – Mary Lamberton Becker

“As for the future, it remains unwritten. Anything can happen, and often we are wrong. The best we can do with the future is prepare and savor the possibilities of what can be done in the present.” – Todd Kashdan

“Even if you are on the right track, you’ll just get run over if you just sit there.” – Will Rogers

“Sometimes the path to higher self-esteem is lonely and frightening. We cannot fully know in advance how much more satisfying our lives will be. But the more we are willing to experience and accept the many aspects of who we are, the richer our inner worlds, the greater our resources, the more appropriate we feel to the challenges and opportunities of life.” – Nathaniel Branden

“Listen. I wish I could tell you it gets better. But, it doesn’t get better. You get better.” – Joan Rivers

“This life is ironic: for it takes pain to discover pleasure; it takes sadness to know happiness; it takes war to value peace; and it takes hatred to treasure love.” – Emmanuel Aghado

“In life there will be pain, suffering, ugliness, but let us be grateful for the beauty, blessing, and miracle of life.” – Debasish Mridha

“I suppose the test of one’s decency is how much of a fight one can put up after one has stopped caring, and after one has found that one can never please the people they wanted to please.” – Willa Cather

“No one will come and save you. No one will come riding on a white horse and take all your worries away. You have to save yourself, little by little, day by day.” – Charlotte Eriksson

“We all have our times of anger. It’s when we hang onto that anger it becomes a poison to our heart and mind.” – Ron Baratono

“For those who have known pain and suffering and wondered why it all happened…I don’t know the reasons why, I just know that you must keep believing in hope and eventually you will get through.” – Heather Wolf

“Your emotions are meant to fluctuate, just like your blood pressure is meant to fluctuate. It’s a system that’s supposed to move back and forth, between happy and unhappy. That’s how the system guides you through the world.” – Daniel Todd Gilbert

“When you’ve spent your whole life not being good enough, it takes time to let yourself believe that you finally are. Self-worth isn’t a switch that flips inside you. It’s a daily struggle not to sabotage your own success.” – Julie Johnson

“Emergencies have always been necessary to progress. It was darkness which produced the lamp. It was fog that produced the compass. It was hunger that drove us to exploration. And it took a depression to teach us the real value of a job.” – Victor Hugo

“It will never rain roses: when we want to have more roses, we must plant more roses.” – George Eliot

---

General Statement: *discusses general concepts or situations in life, often in an observational manner, so as to guide cognitions, mindsets, or perspectives of the world to be more positive, thus promoting wellbeing without imposing on the individual*

“Every person on this earth is full of great possibilities that can be realized through imagination, effort, and perseverance.” – Scott Barry Kaufman

“Surrendering is not giving up – it is gaining strength.” – Grace Sara

“Beauty is sometimes born of pain.” – Sheri L. Swift

“Pain is pain…Just because one person’s problem is less traumatic than another’s doesn’t mean they’re required to hurt less.” – J.A. Redmerski

“One’s dignity may be assaulted, vandalized and cruelly mocked, but it can never be taken away unless it is surrendered.” – Michael J. Fox

“Obstacles are those frightful things you see when you take your eyes off your goal.” – Henry Ford

“Every individual is an expression of the whole realm of nature, a unique action of the total universe.” – Alan Watts

“The world’s greatest achievers have been those who have always stayed focused on their goals and have been consistent in their efforts.” – Roopleen

“‘Healthy’ and ‘diseased’…are often subjective judgments that society makes for its own purposes.” – Naomi Wolf

“True beauty comes from within and no one can ever change it.” – Katrina Denise

“Every beautiful thing in the world is rooted in some kind of pain.” – Bryant McGill

“Pain is a better teacher than pleasure. Poverty is a better teacher than prosperity. Failure is a better teacher than success. Life is a better teacher than university.” – Matshona Dhliwayo

“We are spiritual beings – indestructible, immaterial and safe.” – Donna Goddard

“Being liked or not, having company or not, being understood or not, being acknowledged or not are not issues of concern on the spiritual path.” – Donna Goddard

“Your body is the piece of the universe you’ve been given; as long as you have a pulse, it presents you with an ongoing shower of immediate sensate experiences.” – Geneen Roth

“The good life is a process, not a state of being.” – Carl Rogers

“It is more important to know where you are going than to get there quickly.” – Socrates

“In the story of the Ugly Duckling, when did the ugly Duckling stop feeling Ugly? When he realized that he was a Swan. Each of us has something Special, a Swan of some sort, hidden inside somewhere. But until we recognize that it’s there, what can we do but splash around, treading water?” – Benjamin Hoff

“Learning to love yourself is like learning to walk – essential, life-changing, and the only way to stand tall.” – Vironika Tugaleva

“Starting your day with anger is like locking yourself in jail. The outside world is enjoying life, while you’re locked in your own frustration.” – Ron Baratono

“Nestled in the valley of darkness, in the deepest depths of depression, are the priceless gems of; creativity, intuition and sensitivity. The trick is learning how to navigate the dark, so these precious gems can be unearthed and their beauty beheld.” – Jaeda DeWalt

“I find people with depression are like a broken clock, and clock maker after clock maker will try to fix you, but sometimes time itself is the answer.” – Isaac Andrews

“Joy of harvest belongs only to those who ensure the pain of sowing.” – Sunday Adelaja

“After the rain, the sun will reappear. There is life. After the pain, the joy will still be here.” – Walt Disney Company

“Too often fear is fiction madly running amuck, all the while madly tracking ‘muck’ across the floor of fact.” – Craig D. Lounsbrough

“Just as a star can’t be seen behind a cloud, you can never shine and be seen behind the clouding of your insecurities.” – Dr. Jacinta Mpalyenkana

“Where we fall are the stepping-stones for our journey.” – Lolly Daskal

“There will always be more growing to do. That is the beauty of life. There is always some new opportunity to do something new, to make something old better, to chuck out something useless, to transform something into something else. It's important to spend just as much time seizing these opportunities as appreciating the lessons they teach you and the person you become from seizing them.” – Vironika Tugaleva

“When failure is hard to take, pain becomes unbearable and you don’t see any way out. Hang on there, it’s the darkest hour that indicates the advent of light.” – Evan Smith

“Depression is a sign of strength – because it means no matter how weak your mind might be to you, your heart is still strong enough to feel.” – Emma Hart

“The opposite of depression isn’t feeling happy but being fully alive, however painful.” – Gwyneth Lewis

“Problems come to our life not to give us pain, but to make us humble, kind, strong and fearless.” – Debasish Mridha

“The pain of problems is a call to find solutions rather than a reason for unhappiness and inaction, so it’s silly, pointless, and harmful to be upset at the problems and choices that come at you (though it’s understandable).” – Ray Dalio

“Every pain, addiction, anguish, longing, depression, anger or fear is an orphaned part of us seeking joy, some disowned shadow wanting to return to the light and home of ourselves.” – Jacob Nordby

“A single thought can shift your entire world.” – Alaric Hutchinson

“Right on the other side of our fears resides hope and happiness. We get stuck in our lives afraid to walk over there when it’s a much kinder place.” – Ron Baratono

“We all have anger in our hearts at times from past situations or even present. These thoughts hurt one’s self more than anything. Without the effort of washing those thoughts from our minds there can be no inner peace.” – Ron Baratono

“Your actions are viewed by observers through prisms shaped by their own interpretations, and sometimes misinterpretations. All you can do is strive to be your best self. Let the chips fall where they may.” – Cathryn Louis

“As change is a constant – as our houses, our money, our friends, our things and even our lovers can always disappear – the one thing that we will always remain the closest to, will be our minds and our bodies.” – Lauren Klarfeld

“Because of self-doubt, the fear of failure, or laziness, most people usually bite off way less than they can chew.” – Mokokoma Mokhonoana

“For whatever it’s worth, I believe we’re born imperfect, and perfection, whatever that may be, is unattainable by us mere humans.” – Liza M. Weimer

“Of all the judgments we pass, none is as important as the one we pass on ourselves.” – Nathaniel Branden

“If we lack either self-awareness or confidence in who we are, we run the risk of allowing others to define who we are or who we should be.” – Lisa Locke

“One of the greatest lies is to believe that we don’t have value. One of the greatest mistakes is to act on that belief.” – Craig D. Lounsbrough

“In evaluating ourselves, we tend to be long on our weaknesses and short on our strengths.” – Craig D. Lounsbrough

“Self Hate: The deadliest ‘dis-ease’ experienced by wounded souls.” – T.F. Hodge

“The problem arises when shame kicks in and we aren’t able to view our flaws, limitations, and vulnerabilities in a patient, self-loving way.” – Harriet Lernet

“Far too often we allow the hurtful words of others to define us.” – Eddie Capparucci

“Out of fear, out of the desire for approval, out of misguided notions of duty, people surrender themselves – their convictions and their aspirations – every day. There is nothing noble about it. It takes far more courage to fight for your values than to relinquish them.” – Nathaniel Branden

“To honor the self is to be willing to think independently, to live by our own mind, and to have the courage of our own perceptions.” – Nathaniel Branden

“Saying thanks to the world, and acknowledging your own accomplishments, is a great way to feel good and stay positive.” – Rachel Robin

“A low self-image is usually not based upon facts; it’s mismanaged memory.” – Orrin Woodward

“To turn your dreams into reality, all your resources, efforts and concentration should be aligned in the same direction.” – Roopleen

“Nothing is better for self-esteem than survival.” – Martha Gellhorn

“To the logician all things should be seen exactly as they are, and to underestimate one’s self is as much a departure from truth as to exaggerate one’s own powers.” – Sherlock Holmes

“The greatest thing in the world is to know how to belong to oneself.” – Michel de Montiagne

“We can regret less and be grateful for what is good in life.” – Barry Schwartz

“Happiness is not out there for us to find. The reason that it’s not out there is that it’s inside us.” – Sonja Lyubomirsky

“Vulnerability is not weakness. And that myth is profoundly dangerous.” - Brené Brown

“The highest and most beautiful things in life are not to be heard about, nor read about, nor seen but, if one will, are to be lived.” – Soren Kierkegaard

“It is not primarily our physical selves that limit us but rather our mindset about our physical limits.” – Ellen J. Langer

“Everything can be taken from a man but…the last of the human freedoms – to choose one’s attitude in any given set of circumstances.” – Viktor Frankl

“Probably the biggest insight…is that happiness is not just a place, but also a process…Happiness is an ongoing process of fresh challenges, and…it takes the right attitudes and activities to continue to be happy.” – Ed Diener

“To finally recognize our own invisibility is to finally be on the path toward visibility.” – Yamada

“If our dreams weren’t already real within us, we could not even dream them.” – Gloria Steinem

“There is that gnawing feeling that we are far more than what we believe ourselves to be. Maybe it’s time to believe the gnawing.” – Craig D. Lounsbrough

“We are always immeasurably bigger than the little person we’ve too often doomed ourselves to be.” – Craig D. Lounsbrough

“Unless we can predict the future, we shouldn’t be worrying about things that haven’t happened yet.” – Ron Baratono

“If you feel anxiety or depression, you are not in the present. You are either anxiously projecting the future or depressed and stuck in the past. The only thing you have any control over is the present moment; simple breathing exercises can make us calm and present instantly.” – Tobe Hanson

“I think we all keep irritating situations in our head too long, until we finally realize it’s taking up valuable space.” – Ron Baratono

“Regrets are a terrible thing to live with but, if we take a good look at them, some are not regrets at all, they’re situations that taught us a valuable lesson. Don’t be so hard on yourself, it’s not a perfect world.” – Ron Baratono

“Happiness isn’t about pretending there’s no pain. It’s about accepting the pain as a part of healing and doing your best to nurse your own wounds with love and patience.” – Vironika Tugaleva

“The pain of the journey should challenge you to make change, the vision of what could be drives you forward.” – Tony Curl

“We do not need magic to transform our world. We carry all the power we need inside ourselves already.” – J.K Rowling

“Energy and persistence alter all things.” – Benjamin Franklin

“A mind in peace is heaven; a mind in pieces is hell.” – K. Bhujang Shetty

“The keenest sorrow is to recognize ourselves as the sole cause of all our adversities.” – Sophocles

“Perhaps the only limits to the human mind are those we believe in.” – Willis Harman

“Remember that the happiest people are not those getting more, but those giving more.” – H. Jackson Brown, Jr.

“The miracle is this: the more we share the more we have.” – Leonard Nemoy

“Freedom lies in being bold.” – Robert Frost

“Wisdom is knowing what to do next, skill is knowing how to do it, and virtue is doing it.” – David Starr Jordan

“Adopt the pace of nature: her secret is patience.” – Ralph Waldo Emerson

“It’s not the size of the dog in the fight, it’s the size of the fight in the dog.” – Mark Twain

“Serenity isn’t freedom from the storm, but peace within the storm.” – Unknown

“Great things are done by a series of small things brought together.” – Vincent Van Gogh

“Who can say where the road goes, Where the day flows? Only time…” – Enya

“It always seems impossible until it’s done.” – Nelson Mandela

“Greatness lies, not in being strong, but in the right using of strength.” – Henry Ward Beecher

“All that we are is the result of what we have thought.” – Buddha

“Life is really simple, but we insist on making it complicated.” – Confucius

“We must build dikes of courage to hold back the flood of fear.” – Martin Luther King, Jr.

“The longest journey begins with a single step, not with the turn of the ignition key.” – Edward Abbey

“There is nothing in a caterpillar that tells you it’s going to be a butterfly.” – Buckminster Fuller

“The hard must become habit. The habit must become easy. The easy must become beautiful.” – Doug Henning

“Hope is being able to see that there is light despite all of the darkness.” – Desmond Tutu

“All the flowers of tomorrow are in the seeds of today.” – Anonymous

“You miss 100 percent of the shots you never take.” – Wayne Gretzky

“Where there is love there is life.” – Gandhi

“With love and patience, nothing is impossible.” – Soka Gakkai

“Life is like a ten-speed bike. Most of us have gears we never use.” – Schultz

“It is not the mountain we conquer, but ourselves.” – Sir Edmund Hillary

“The most wasted of all days is one without laughter.” – E.E. Cummings

“There is more to life than increasing its speed.” – Gandhi

“When it is dark enough, you can see the stars.” – Ralph Waldo Emerson

“The only impossible journey is the one you never begin.” – Tony Robbins

“Sometimes the most urgent thing you can possibly do is take a complete rest.” – Asheligh Brilliant

“Happiness is not a state to arrive at, but a manner of traveling.” – Margaret Lee Runbeck

“The greatest pleasure in life, is doing the things people say we cannot do.” – Walter Bagehot

“The future belongs to those who believe in the beauty of their dreams.” – Eleanor Roosevelt
